# Differential distribution and genetic determination of eccrine sweat glands and hair follicles in the volar skin of C57BL/6 mice and SD rats

**DOI:** 10.1186/s12917-022-03416-z

**Published:** 2022-08-16

**Authors:** Zixiu Chen, Junhong Zhao, Yongjing Yan, Lei Zhang, Lijie Du, Xiang Liu, Manxiu Cao, Cangyu Wang, Yue Tang, Haihong Li

**Affiliations:** 1 Jinzhou Medical University Graduate Training Base, Department of Wound Repair and Dermatologic Surgery, Taihe Hospital, Hubei University of Medicine, Shiyan, Hubei Province China; 2 Department of Wound Repair and Dermatologic Surgery, Taihe Hospital, Hubei University of Medicine, Shiyan, Hubei Province China; 3 Hubei Clinical Medical Research Center of Cord Blood Hematopoietic Stem Cells, Taihe Hospital, Hubei University of Medicine, Shiyan, Hubei Province China; 4 Mental Health Center, Taihe Hospital, Hubei University of Medicine, Shiyan, Hubei Province China; 5 Department of Wound Repair; Institute of Wound Repair and Regeneration Medicine, Southern University of Science and Technology Hospital, Southern University of Science and Technology School of Medicine, Shenzhen, China

**Keywords:** Eccrine sweat glands, Hair follicles, Footpads, Inter-footpads, Volar skin, *LHX2*, *En1*, C57BL/6 mice, SD rats

## Abstract

**Background:**

Eccrine sweat glands (ESGs) and hair follicles (HFs) are the prominent skin appendages regulating human body temperature. C57BL/6 mice and Sprague–Dawley (SD) rats are the most commonly used model animals for studying ESGs and HFs. Previous studies have shown the distribution of ESGs and HFs in volar hindfeet of C57BL/6 mice, but there are few or no reports on the distribution of ESGs and HFs in volar forefeet of C57BL/6 mice and volar feet of SD rats. Here, we investigated the differential distribution and genetic determination of ESGs and HFs in the volar skin of C57BL/6 mice and SD rats through gross observation, iodine-starch sweat test, double staining with Nile Blue A and Oil Red O, hematoxylin and eosin (HE) staining, double immunofluorescence staining of LIM Homeobox 2 (LHX2)/Na^+^-K^+^-ATPase α1(NKA) or LHX2/Na^+^-K^+^-2Cl^－^ cotransporter 1 (NKCC1), and qRT-PCR detection of ESG-related gene *Engrailed 1* (*En1*) and HF-related gene *LHX2*.

**Results:**

The results showed ESGs but no HFs in the footpads of C57BL/6 mice and SD rats, both ESGs and HFs in the inter-footpads (IFPs) of C57BL/6 mice, and neither ESGs nor HFs in the IFPs of SD rats. The relative quantitative change in *En1* was consistent with the differential distribution of ESGs, and the relative quantitative change of *LHX2* was consistent with the differential distribution of HFs.

**Conclusion:**

C57BL/6 mice and SD rats had their own characteristics in the distribution of ESGs and HFs in the volar skin, and researchers should choose mice or rats, and even forefeet or hindfeet as their research object according to different purposes. The study provides a basis for selection of optimal animal models to study development, wound healing and regeneration of skin appendages.

**Supplementary Information:**

The online version contains supplementary material available at 10.1186/s12917-022-03416-z.

## Background

Hair follicles (HFs), sebaceous glands, and eccrine sweat glands (ESGs) are skin appendages that function in protection, excretion, and thermoregulation [1–3]. ESGs and HFs coexist in most human skin, but ESGs are only present in the palms and soles [4, 5]. Maintaining a stable internal body temperature is essential for mammal survival [6]. In humans, thermoregulation is achieved by evaporating water secreted by the ubiquitous ESGs, and short and sparse hairs contribute to efficient sweat evaporation and skin cooling [4, 7, 8]. The increase in ESG density, and the decrease in HF density and size, are the results of adaptive evolution in humans, allowing them to regulate body temperature more effectively [7, 9]. Both ESGs and HFs originate from the ectoderm, but many of their developmental signals are spatiotemporal antagonistic [10]. Therefore, it is essential to clarify the molecular programs that control the development, pattern, and evolution of ESGs and HFs.

Sprague-Dawley (SD) rats and C57BL/6 mice are the most commonly used model animals for studying ESGs and HFs [11–14]. Previous studies have shown that ESGs are limited to the volar skin of rats and mice, and serve a role in traction during locomotion [15, 16]. Further studies on the hindfeet of C57BL/6 mice have shown that there are ESGs and HFs in the inter-footpads (IFPs), but only ESGs in the footpads [9]. There are few studies on the distribution of ESGs and HFs in the volar forefeet of mice, although it is generally accepted that there are only ESGs and no HFs [10, 17]. To date, there are no reports on the distribution of ESGs and HFs in the volar skin of SD rats. Here, we investigated and compared the differential distribution and genetic determination of ESGs and HFs in the volar skin of C57BL/6 mice and SD rats, the results of which will provide a basis for selection of optimal animal models to study the development, wound healing and regeneration of skin appendages.

## Results

### Differential distribution of ESGs and HFs in the volar skin of C57BL/6 mice and SD rats

**Fig. 1:**
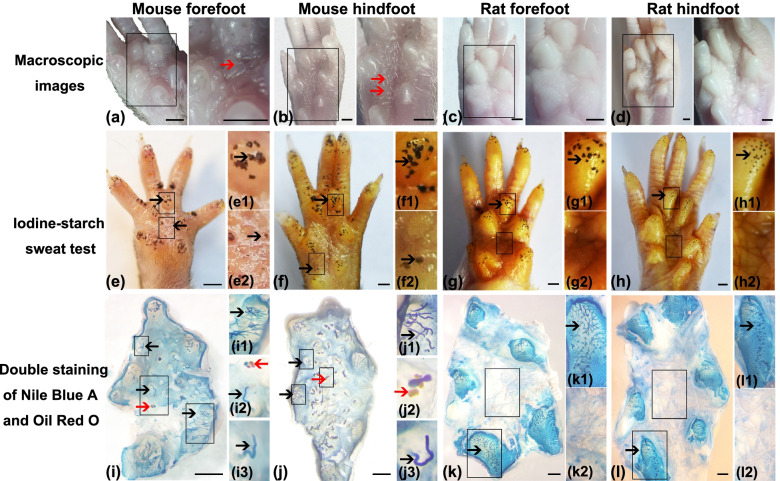
Distribution of ESGs and HFs in the volar skin of C57BL/6 mice and SD rats. **a**–**d** Gross phenotype of the volar skin of mouse forefoot (a, *n* = 60), mouse hindfoot (b, *n* = 20), rat forefoot (c, *n* = 20), and rat hindfoot (d, *n* = 20). The right panels show magnified views of the boxes in the left panels. 30 mice and 10 rats were used. **e**–**h** Representative images of iodine-starch sweat test on mouse forefoot (e, *n* = 20), mouse hindfoot (f, *n* = 20), rat forefoot (g, *n* = 20), and rat hindfoot (h, *n* = 20). 10 mice and 10 rats were used. Black dots represent sweating spots. (e1, f1, g1, h1) and (e2, f2, g2, h2) are magnified views of the footpads and IFPs in the boxed area shown in (e, f, g, h), respectively. **i**–**l** Representative images of epidermal preparations from the volar skin of mouse forefoot (i, *n* = 10), mouse hindfoot (j, n = 10), rat forefoot (k, *n* = 10), and rat hindfoot (l, *n* = 10). 5 mice and 5 rats were used. (i1, j1, k1, l1) and (i2, i3, j2, j3, k2, k3, l2, l3) are magnified views of the footpads and IFPs in the boxed area shown in (**i**, **j**, **k**, **l**), respectively. ESG ducts stained with Nile Blue A are the elongated blue tubes emerging inside the footpads or IFPs. Oil Red O staining marks HF-related sebaceous glands (red). Red arrows indicate HFs, and black arrows indicate ESGs. Scale bar = 1 mm. Abbreviations: IFPs, inter-footpads; HFs, hair follicles; ESGs, eccrine sweat glands; SD, Sprague-Dawley

In gross morphology, the distribution of ESGs and HFs in the volar skin of C57BL/6 mice and SD rats was detected by macroscopic observation, iodine-starch sweat test, and double staining of Nile Blue A and Oil Red O. The macroscopic observation was used to determine the distribution of HFs (Fig. 1a−d). Macroscopically, hair was observed in the fore- and hind-IFPs of C57BL/6 mice, but not in the footpads of C57BL/6 mice, and the footpads and IFPs of SD rats (Fig. 1a–d). The iodine-starch sweat test was used to identify the distribution of ESGs (Fig. 1e–h). The black dots represent sweat droplets secreted by ESGs. The sweat droplets were present in C57BL/6 mouse footpads (Fig. 1e1, f1) and IFPs (Fig. 1e2, f2) and SD rat footpads (Fig. 1 g1, h1), but not in SD rat IFPs (Fig. 1 g2–h2). Nile Blue A was used to label the sweat ducts and Oil Red O was used to label the sebaceous glands in the pilosebaceous unit. Double staining with Nile Blue A and Oil Red O showed that C57BL/6 mice and SD rats had ESGs but no HFs in the footpads (Fig. 1i1, j1, k1, l1), and C57BL/6 mice had both ESGs and HFs in the IFPs (Fig. 1i2, i3, j2, j3), but SD rats had neither ESGs nor HFs in the IFPs (Fig. 1 k2, l2).

**Fig. 2:**
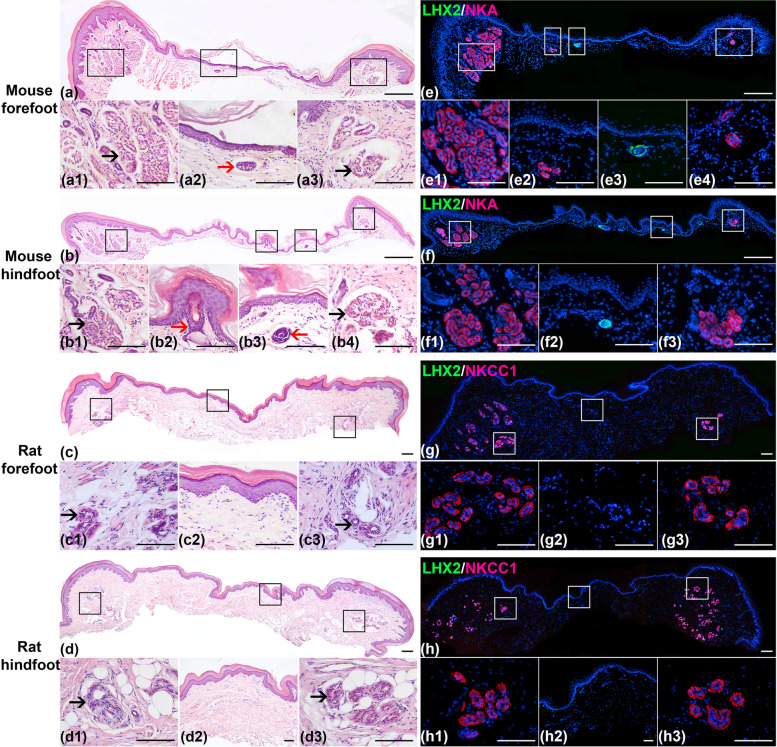
Histological staining of the volar skin of C57BL/6 mice and SD rats. The slightly raised structures on both sides of the tissue sections are the footpads and the relatively flat structures in the middle of the tissue sections are IFPs. **a**–**d** HE staining of the volar skin in mouse forefoot (**a**), mouse hindfoot (**b**), rat forefoot (**c**), and rat hindfoot (**d**). (a1, a3, b1, b4, c1, c3, d1, d3) and (a2, b2, b3, c2, d2) are magnified views of the footpads and IFPs in the boxed area shown in (**a**–**d**), respectively. Red arrows indicate HFs, and black arrows indicate ESGs. **e**–**h** Double immunofluorescent staining of LHX2/NKA or LHX2/NKCC1 of the volar skin in mouse forefoot (**e**), mouse hindfoot (**f**), rat forefoot (**g**), and rat hindfoot (**h**). LHX2, HF marker (green). NKA and NKCC1, ESG markers (red). The cell nuclei were counterstained with DAPI (blue). (e1, e4, f1, f3, g1, g3, h1, h3) and (e2, e3, f2, g2, h2) are magnified views of the footpads and IFPs in the boxed area shown in (**e**–**h**), respectively. 5 mice and 5 rats were used. The number of mouse forefoot, mouse hindfoot, rat forefoot and rat hindfoot were 10, respectively. Scale bar, 100 μm (a, b, c, e, f, g, h lower panels), 300 μm (a, b, e, f, g, h upper panels). Abbreviations: NKA, sodium potassium ATPase α1; NKCC1, Na^+^-K^+^-2Cl^−^ cotransporter 1; LHX2, LIM Homeobox 2

In the micromorphology, hematoxylin and eosin (HE) staining and double immunofluorescence staining were used (Fig. 2a–h). HE staining showed that both ESGs (See Supplementary Fig. 1) and HFs (Fig. 2a2, b2, b3) were present in the IFPs of C57BL/6 mice, only ESGs were present in the footpads of C57BL/6 mice and SD rats (Fig. 2a1, a3, b1, b4, c1, c3, d1, d3), but no ESGs and HFs were present in the IFPs of SD rats (Fig. 2c2, d2). Double immunofluorescence staining of Na ^+^-K^ +^-ATPase α1 (NKA)/LIM Homeobox 2 (LHX2) and Na^ +^-K^ +^-2Cl^－^ cotransporter 1 (NKCC1)/LHX2 showed that HF-specific marker, LHX2, was detected only in the IFPs of C57BL/6 mice (Fig. 2e3, f2), whereas ESG-specific markers, NKA or NKCC1, were detected in the footpads and IFPs of C57BL/6 mice, and the footpads of SD rats (Fig. 2g–h).

### Differences in the number of HFs and ESGs in the volar skin of C57BL/6 mice and SD rats

**Fig. 3:**
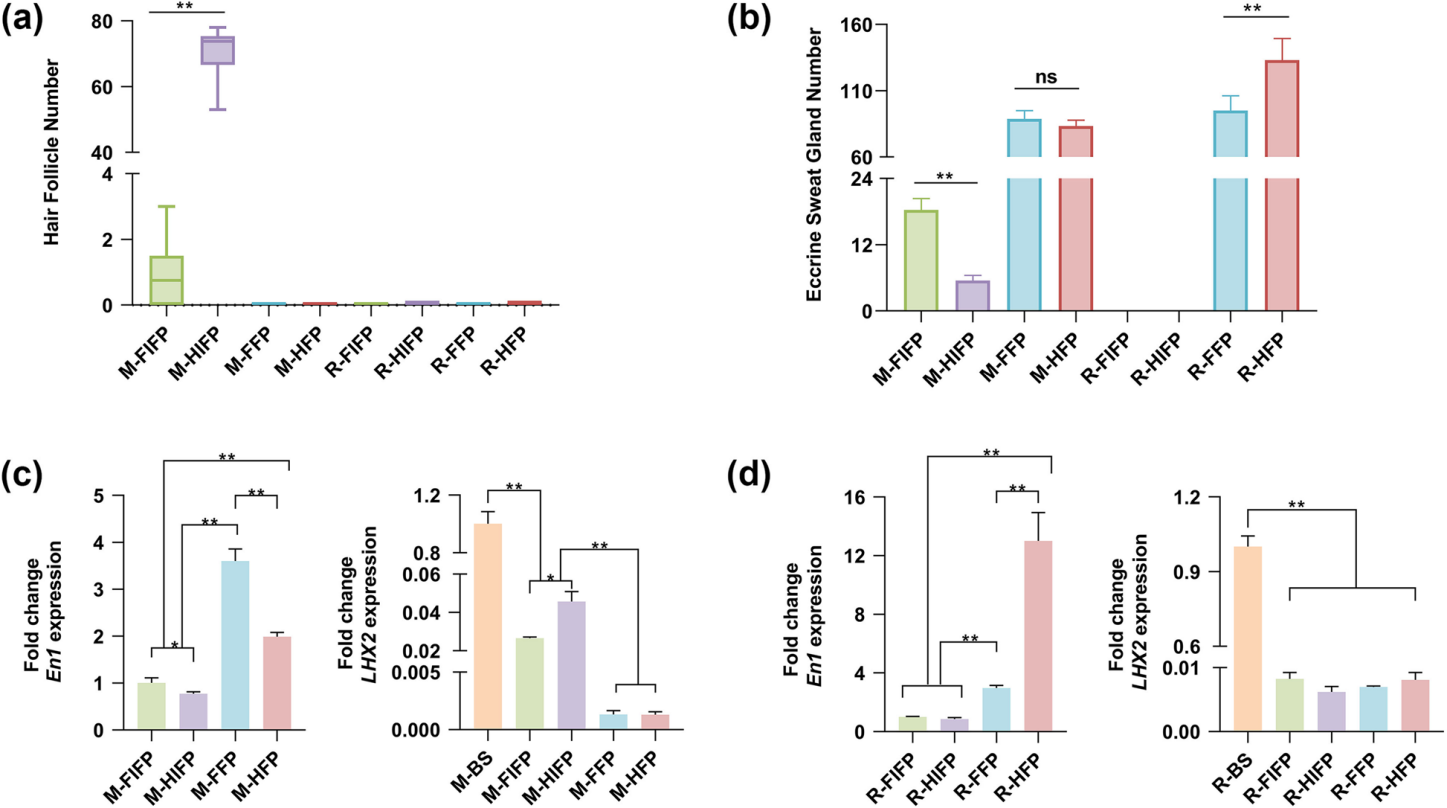
The number and gene expression of ESGs and HFs. **a** Quantification of HFs in the footpads and IFPs of mouse forefoot (*n* = 60), mouse hindfoot (*n* = 20), rat forefoot (*n* = 20), and rat hindfoot (*n* = 20). 30 mice and 10 rats were used. The median and inter-quartile ranges are plotted. **b** Quantification of ESGs in the footpads and IFPs of mouse forefoot (*n* = 20), mouse hindfoot (*n* = 20), rat forefoot (*n* = 20), and rat hindfoot (*n* = 20). 10 mice and 10 rats were used. Values are presented as mean ± standard deviation. **a**–**b** Every value represents average number of ESGs and HFs in the footpads or IFPs of a single animal’s two forefeet or two hindfeet. **c**–**d** qRT-qPCR: differential expression of *En1* in four tissues of mouse or rat (FIFP, HIFP, FFP, HFP). Differential expression of *LHX2* in five tissues of mouse or rat (BS, back skin, as a positive control; FIFP, HIFP, FFP, HFP). For all qRT-PCR analyses, gene expression was normalized to the reference gene (GAPDH). Values are reported as mean ± standard deviation. **a**-**d** Kruskal-Wallis (**a**), Welch’s ANOVA (**b**), one-way ANOVA tests (**c**, **d**) were used to test for differences among multiple groups of the data. *p < 0.05, **p < 0.01. “ns” means no statistical significance. Abbreviations: BS, back skin; FIFP, fore inter-footpad; HIFP, hind inter-footpad; FFP, fore-footpad; HFP, hind-footpad; M, mouse; R, rat

In the volar skin of C57BL/6 mice and SD rats, HFs were only present in the IFPs of C57BL/6 mice. The number of HFs in the fore-IFPs of C57BL/6 mice was significantly less than that in the hind-IFPs (Fig. 3a). In mouse forefeet, about 63.3% of IFPs had HFs, and each IFP had 0.75 (median) HFs, whereas in the hindfeet, all IFPs had HFs, and the number of HFs per IFP was as high as 73.75 (Fig. 3a). ESGs were present in the footpads of C57BL/6 mice and SD rats and IFPs of C57BL/6 mice. In C57BL/6 mice, the number of ESGs in the fore-footpads (mean 88.65) was similar to that in the hind-footpads (mean 83.30), but the number of ESGs in the fore-IFPs (mean 18.3) was approximately three times that in the hind-IFPs (mean 5.5) (Fig. 3b). In SD rats, the number of ESGs in the fore-footpads (mean 95.05) was lower than that in the hind-footpads (mean 133.1) (Fig. 3b). ESGs were densely distributed in the footpads of C57BL/6 mice and SD rats, and scattered in the IFPs of C57BL/6 mice.

### The genetic determination of ESGs and HFs in the volar skin of C57BL/6 mice and SD rats

The expression of ESG-related gene *Engrailed 1* (*En1*) and HF-related gene *LHX2* was detected by qRT-PCR, and the relative quantitative change was analyzed. In C57BL/6 mice, the expression of *En1* was highest in the fore-footpads, followed by the hind-footpads, fore-IFPs, and hind-IFPs (Fig. 3c). The expression of *En1* was significantly different between footpads and IFPs, between fore-footpads and hind-footpads, and between fore-IFPs and hind-IFPs (Fig. 3c). In SD rats, the expression of *En1* was highest in the hind-footpads, followed by the fore-footpads and IFPs (Fig. 3d). There were significant differences in *En1* expression between the footpads and IFPs and between the fore-footpads and the hind-footpads (Fig. 3d). In the volar skin of C57BL/6 mice, *LHX2* was most expressed in the hind-IFPs, followed by the fore-IFPs, while *LHX2* was barely undetectable in the footpads (Fig. 3c). The expression of *LHX2* in IFPs was still significantly lower than in back skin (Fig. 3c). Compared with back skin, *LHX2* was almost undetectable in the volar skin of SD rats (fore- and hind-footpads, fore- and hind-IFPs) (Fig. 3d).

## Discussion

In the study, we investigated the differential distribution and genetic determination of ESGs and HFs in the volar skin of C57BL/6 mice and SD rats. Previous studies have shown that membrane transport proteins NKCC1 and NKA are related to sweat secretion and reabsorption, and are expressed in the basolateral plasma membrane of secretory coil cells of rat, mouse, and human ESGs [18–20]. Transcription factor En1, a hallmark of ESG-forming epidermis and ESG placodes, plays a pivotal role in the ESG formation [21, 22]. *En1* knockout mice fail to form ESGs but can form HFs [10, 23, 24]. Transcription factor LHX2, a downstream signal that specifies HF fate, is expressed in the early HF placodes, the leading front of invaginating HFs, and the bulges of mature HFs, but not in the absence of HF induction [25, 26]. *LHX2* knockout mice fail to maintain hair characteristics and become bald over time, and the HFs gradually lose the stem cell markers and transform into sebaceous glands [27]. In the study, NKCC1, NKA, and En1 are used as specific markers for ESGs, and LHX2 is used as a specific marker for HFs [18, 22, 28].

We first detect the differential distribution of ESGs and HFs by macroscopic observation, iodine-starch sweat test, double staining with Nile Blue A and Oil Red O, HE staining, and double immunofluorescence staining. Our results show that there are ESGs but no HFs in the footpads of C57BL/6 mice and SD rats, and both ESGs and HFs in the IFPs of C57BL/6 mice, but neither ESGs nor HFs in the IFPs of SD rats. The localization of ESGs and HFs in the hind feet of C57BL/6 mice in our study is consistent with the study by Kamberov et al. [9, 22]. Kamberov et al. showed that the hind-footpads of C57BL/6 mice were densely distributed with ESGs, similar to the glabrous skin of human palms and soles, and the hind-IFPs were interspersed with ESGs and HFs, similar to human hairy skin. However, the distribution of ESGs and HFs in the forefeet of C57BL/6 mice in our study was slightly different from that reported by Kunisada et al. In the study by Kunisada et al., they showed that there was no HFs in the volar forefeet, while in our study, about 63.3% of fore-IFPs had a small number of HFs [17]. The inconsistency may be due to individual differences in C57BL/6 mice, as some of C57BL/6 mice in our study did not have HFs in their fore-IFPs.

It is worth noting that the footpads of SD rats have ESGs but no HFs, which is the same as the footpads of C57BL/6 mice, while the IFPs of SD rats have neither ESGs nor HFs, which is different from the IFPs of C57BL/6 mice. As far as we know, it is the first report on the distribution of ESGs and HFs in the volar skin of rats. Rat footpad skin only supports ESG morphogenesis, whereas rat IFP skin supports neither ESG nor HF morphogenesis. By comparing the differences in DNA, RNA, proteins, and metabolites between the footpads and IFPs of rats, essential information about the development, wound repair, and regeneration of ESGs can be inferred.

Second, to determine the genetic determination of ESGs and HFs in the footpads and IFPs of C57BL/6 mice and SD rats, we examine the mRNA expression levels of ESG-related gene *En1* and HF-related gene *LHX2* by qRT-PCR. The results show that in the footpads and IFPs of C57BL/6 mice and SD rats, the relative quantitative change of *En1* is consistent with the difference distribution of ESGs, and the relative quantitative change of *LHX2* is consistent with the difference distribution of HFs. A previous study showed that change in the level of *En1* activity had different effects on the IFPs and footpads of C57BL/6 mice [9]. The change in *En1* expression had a qualitative effect on the properties of skin appendages in the IFPs, but only had a quantitative effect on the ESGs in the footpads. When *En1* expression was reduced in the footpads, fewer ESGs were formed, but HFs did not replace the ESGs. However, when *En1* expression decreased in the IFPs, fewer ESGs and more HFs were formed, indicating that *En1* levels regulated the relative proportions of ESGs and HFs in the two appendages coexisted regions.

## Conclusions

**Fig. 4:**
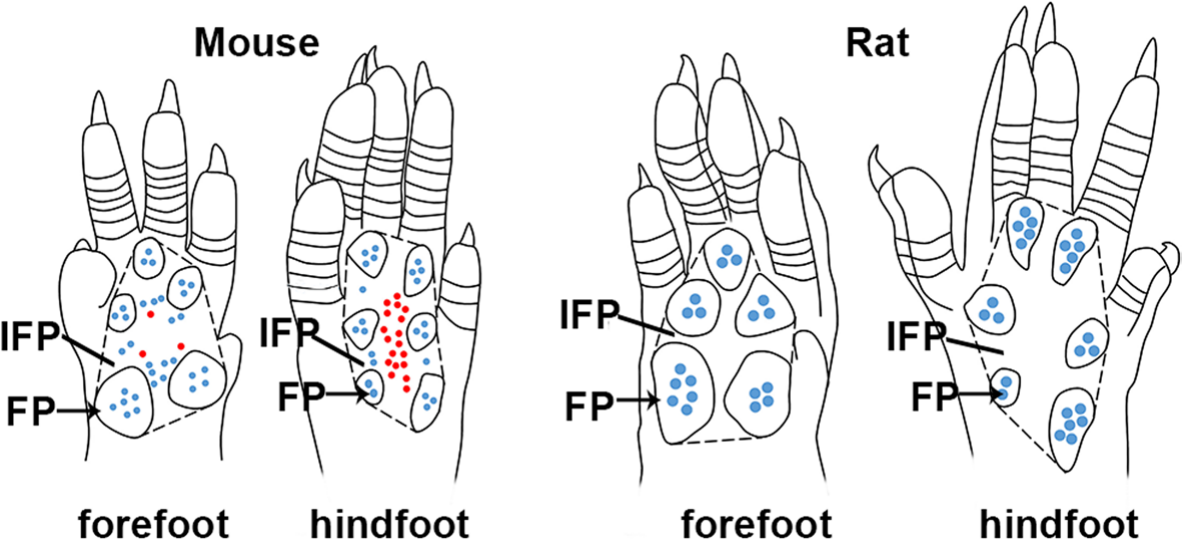
Schematic diagram of the volar skin of C57BL/6 mice and SD rats. Schematic diagram showing the distribution pattern of ESGs and HFs in the volar skin of mouse forefoot, mouse hindfoot, rat forefoot, and rat hindfoot. The volar skin of mouse and rat feet consists of footpads and IFPs. The black dashed lines mark the border of IFPs, excluding footpads. Red dots indicate HFs, blue dots indicate ESGs, and black arrows indicate footpads

In summary, C57BL/6 mice and SD rats have their own characteristic distribution of ESGs and HFs in the volar skin (Fig. 4). In C57BL/6 mice, there are ESGs but no HFs in the footpads, and both ESGs and HFs in the IFPs. In SD rats, there are ESGs but no HFs in the footpads, and neither ESGs nor HFs in the IFPs. Therefore, according to different purposes, researchers should choose mice or rats, and even forefeet or hindfeet as their research object. To address the evolution, pattern, and mechanisms between ESGs and HFs, the volar hindfeet of C57BL/6 mice, especially the hind-IFPs, are preferred; to study the development, wound repair, and regeneration of ESGs, the volar feet of SD rats are the first choice, followed by the forefeet of C57BL/6 mice. Our results will provide a valuable reference for selecting appropriate animal models in future ESG and HF research.

## Materials and methods

### Ethics statement

All animal experiments were approved by Hubei University of Medicine Animal Care and Use Committee (approval number 2020–007), and conducted according to the National Institutes of Health Guide for the Care and Use of Laboratory Animals. All efforts were made to minimize the number of animals and their suffering throughout the experiment.

### Volar skin specimens

The study was carried out in compliance with the ARRIVE guidelines. The SD rats and C57BL/6 mice, 8–10 weeks old, were obtained from the Laboratory Animal Center of Hubei University of Medicine (Shiyan, China). After euthanasia with an overdose of pentobarbital sodium, the forefeet and hindfeet of the rats and mice were removed and photographed on a stereomicroscope equipped with a camera (Olympus SZX2-ILLT, Tokyo, Japan). Next, the volar skin of C57BL/6 mice and SD rats was cut with microdissection scissors and unfolded with the epidermis facing up, and the hair on the volar skin was counted. Two researchers counted the hairs separately, and an average value was reported. Finally, the volar skin specimens were collected, fixed in 4% paraformaldehyde, embedded in paraffin and cut into 5-μm-thickness sections for HE and double immunofluorescence staining.

### Iodine-starch sweat test to detect active ESGs

This assay was modified from a previous study [29]. First, the animals were anesthetized by intraperitoneal injection of 1% pentobarbital sodium, and then 2% (w/v) iodine/ethanol solution was applied to the volar surface of the forefeet and hindfeet. After the surface was dry, 1 g/ml starch/castor suspension was applied. Then sweat secretion was stimulated by intraperitoneal injection of pilocarpine (2.5 mg/kg). Fine black dots appeared on the volar surface within 3–5 min. When the black dots were stable, and no new dots appeared, representative images were taken with a Nikon D7500 camera. Two researchers counted the black dots independently under a stereomicroscope, and an average value was recorded. The number of black dots represents the number of active ESGs. Ten C57BL/6 mice and ten SD rats were used. The number of ESGs in the footpads and IFPs of the two forefeet and two hindfeet of each animal was calculated and analyzed.

### Epidermal preparation and double staining with Nile Blue A and Oil Red O

The epidermal preparation and double staining protocol were performed as previously described with some modifications [23]. Briefly, the volar skin of the forefeet and hindfeet was dissected and incubated in Dispase II (Aladdin D195752, Shanghai, China) at 4 °C for 18-20 h to separate the epidermis from the underlying dermis. Skin appendages, such as ESG ducts, HFs, and HF-associated sebaceous glands, remained attached to the epidermis. Whole-mount epidermal preparations were stained firstly with Oil Red O (Solarbio G1260, Beijing, China) for 10 min, washed in double-distilled water (ddH2O), stained with 0.1% Nile Blue A (Sigma N0766, Saint Louis, USA) for 1 min, and finally stored in ddH2O. The ESG ducts were dyed blue, and HF-associated sebaceous glands were dyed red. Representative images were taken under a stereomicroscope equipped with a camera (Olympus SZX2-ILLT, Tokyo, Japan).

### HE staining

The sections were stained with a HE Staining kit (Solarbio, Beijing, China) according to the manufacturer’s instructions. Representative images were taken with an inverted microscope (Leica DMI4000B, Germany).

### Double immunofluorescence staining of NKCC1/LHX2 or NKA/LHX2

The sections were deparaffinized in xylene, re-hydrated in graded ethanol, immersed in Tris-ethylenediaminetetraacetic acid (EDTA) buffer (10 mM Tris, 1 mM ethylenediaminetetraacetic acid and 0.05% Tween, pH 9.0) for antigen retrieval, and incubated with 5% normal donkey serum to block nonspecific sites. Subsequently, rat sections were incubated with goat anti-NKCC1 (1:200, sc-21545, Santa Cruz Biotechnology, USA) and rabbit anti-LHX2 (1:500, ab184337, Abcam, USA) primary antibodies, then incubated with Cy3-labeled donkey anti-goat IgG (1:500, A0502, Beyotime, China) and Alexa Fluor 488-labeled donkey anti-rabbit IgG (1:400, A21206, Invitrogen, USA) secondary antibodies. Mouse sections were incubated with mouse anti-NKA (1:200, ab7671, Abcam, USA) and rabbit anti-LHX2 primary antibodies, then incubated with Cy3-labeled goat anti-mouse IgG (1:500, A0521, Beyotime, China) and Alexa Fluor 488-labeled donkey anti-rabbit IgG secondary antibodies. Finally, both rat and mouse sections were stained with 4′, 6-diamidino-2-phenylindole (DAPI, C1006, Beyotime, China) and mounted with an anti-fluorescence quenching agent (P0128M, Beyotime, China). Phosphate buffered saline (PBS) was used to rinse the sections between steps. The negative control sections were incubated with a normal serum of the same species instead of the primary antibodies under the same experimental conditions. Representative immunofluorescence images were taken with an inverted fluorescence microscope (Leica DMI4000B, Germany).

### qRT-PCR to detect the gene expression of *En1* and *LHX2*

Eight rats and eight mice were used. The volar skin from the forefeet and hindfeet was dissected respectively under a stereomicroscope, and then the protruding footpads and the flat IFPs were divided with a scalpel. The RNA of the footpads and IFPs was extracted using the RNA-easy Isolation Reagent kit (R701, Vazyme, China), and the RNA was transcribed into cDNA using the Superscript III First-Strand cDNA Synthesis Kit (Vazyme, China). The qRT-PCR analysis of *En1* and *LHX2* was performed in the CFX96™ Real-Time PCR System (Bio-Rad) using ChamQ Universal SYBR green qPCR Master Mix (Q711, Vazyme, China), with three biological replicates. Gene expressions of *En1* and *LHX2* were normalized to the glyceraldehyde-3-phosphate dehydrogenase (GAPDH), and the relative changes in gene expression were analyzed using the 2^-ΔΔCT^ method. The primers are listed in Table 1.

**Table 1:** Primer Sequences of qRT-PCR

Primer names	Primer Sequences (5′-3′)
rat-GAPDH-F	CAGTGCCAGCCTCGTCTCAT
rat-GAPDH-R	AGGGGCCATCCACAGTCTTC
rat-LHX2-F	CTGGTGTGGACAAGACTTCGGATG
rat-LHX2-R	TGAGGGTTGTAGGAGTGCTGGAG
rat-En1-F	CAAGCGTGCCAAGATCAAGAAAGC
rat-En1-R	CCTGGACCGTGGTGGTAGAGTG
mouse-GAPDH-F	TGTTTCCTCGTCCCGTAGA
mouse-GAPDH-R	ATCTCCACTTTGCCACTGC
mouse-LHX2-F	GAATACCCAGCACACTTTAACC
mouse-LHX2-R	CATCGTTCTCGTTACAGCTAAG
mouse-En1-F	CTACTCATGGGTTCGGCTAAC
mouse-En1-R	CTTGTCTTCCTTCTCGTTCTTT

### Statistical analysis

Statistical analysis was performed in SPSS 24.0 or GraphPad Prism 8 software. Because of the small sample size (*n* ≤ 50), the data were checked for normality using the Shapiro-Wilk test. The normal variables were presented as mean ± standard deviation, and the non-normal variables were reported as median and interquartile range. For comparisons among multiple groups, when the data were normally distributed, one-way analysis of variance (ANOVA) was used for homogeneous variances, followed by least significant difference (LSD) *t* test (qRT-PCR data), and Welch’s ANOVA was used for non-homogeneous variances, followed by Dunnett’s T3 test (ESG number data). When the data were not normally distributed, the Kruskal-Wallis test was used, followed by Pairwise Comparisons (HF number data). A value of *P* < 0.05 was considered significant. *p < 0.05, **p < 0.01. “ns” means no statistical significance.

## Supplementary Information

**Additional file 1.**

## Data Availability

All data generated or analyzed during this study are included in this published article.

## Abbreviations

HFs: Hair follicles
ESGs: Eccrine sweat glands
IFPs: Inter-footpads
NKA: Sodium potassium ATPase α1
NKCC1: Na^+^-K^+^-2Cl^−^ cotransporter 1
LHX2: LIM Homeobox 2
GAPDH: Glyceraldehyde-3-phosphate dehydrogenase
ANOVA: analysis of variance
