# Supplementary material for: Differential distribution and genetic determination of eccrine sweat glands and hair follicles in the volar skin of C57BL/6 mice and SD rats
Source: BMC Vet Res. 2022 Aug 16;18:316. doi: 10.1186/s12917-022-03416-z (PMC9380334; doi:10.1186/s12917-022-03416-z)
Supplement: Supplementary file 1 — Additional file 1. [file 12917_2022_3416_MOESM1_ESM.pdf]

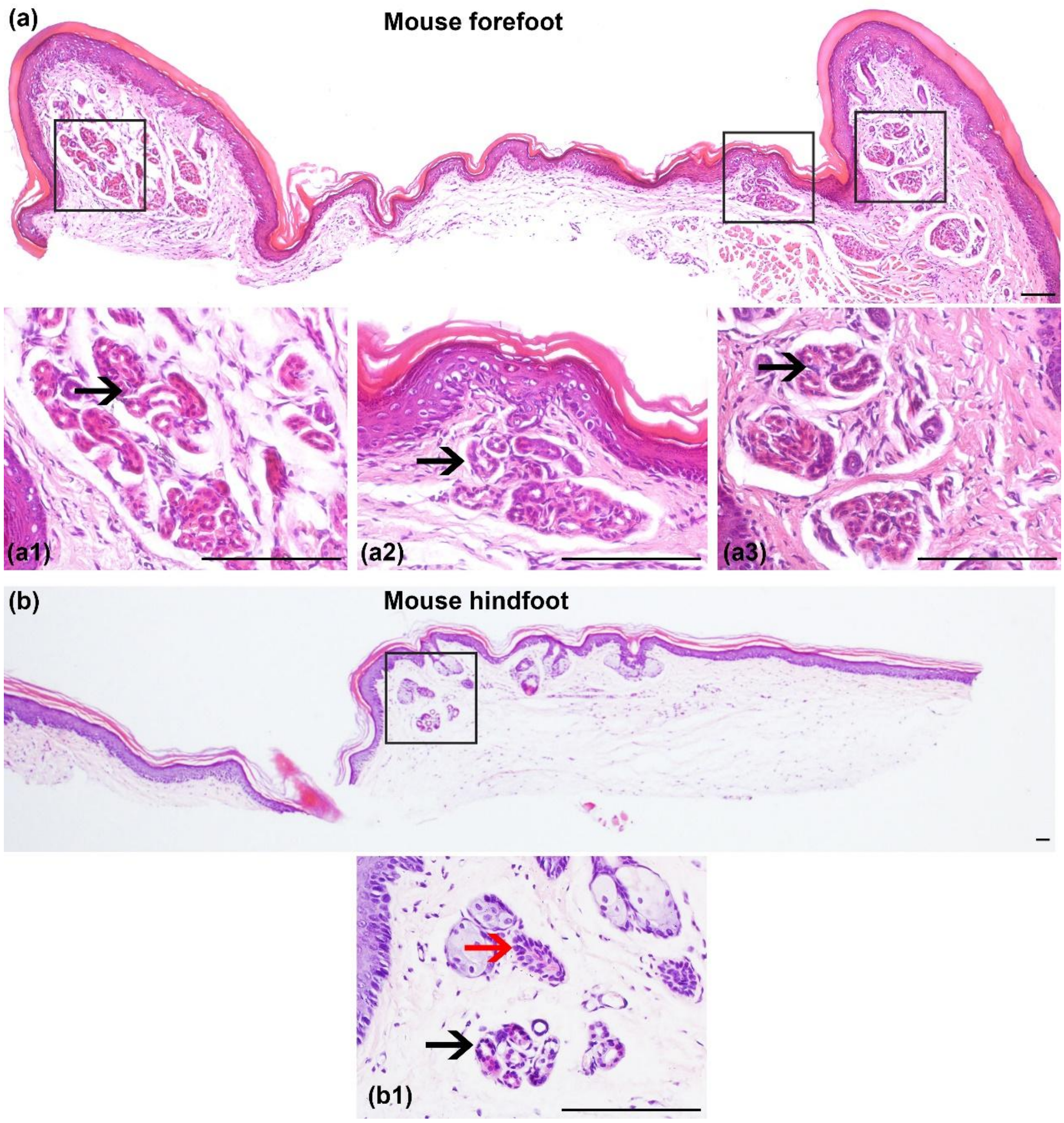

Supplementary Fig. 1 HE staining of the volar skin of C57BL/6 mice. (a) HE staining of the volar skin in mouse forefoot. (a1, a3) The ESGs of mouse footpads. (a2) The ESGs of mouse IFPs. (a1, a2, a3) The magnified views of the boxes in (a). (b) HE staining of the IFPs in mouse hindfoot. (b1) The ESGs and HFs of mouse IFPs. (b1) The magnified view of the box in (b). Red arrow indicates HFs, and black arrow indicates ESGs. Scale bar, 100μm.
